# Editorial: The role of movement variability in motor control and learning, analysis methods and practical applications

**DOI:** 10.3389/fpsyg.2023.1260878

**Published:** 2023-08-03

**Authors:** Francisco J. Moreno, Carla Caballero, David Barbado

**Affiliations:** ^1^Sport Sciences Department, Miguel Hernández University of Elche, Elche, Spain; ^2^Alicante Institute for Health and Biomedical Research (ISABIAL), Alicante, Spain

**Keywords:** adaptation, complexity, coordination, capabilities, dexterity, impairment, movement dynamics

Human movement arises from the intricate interplay among various independent components comprising the human body and their interaction with the surrounding environment. This interaction aims to attain successful motor execution by selecting an appropriate configuration of degrees of freedom (DoF) among all the many available, contingent upon the individual's proficiency and environmental inputs (Gray, 2020). Movement variability has been linked with a CNS mechanism that endows the system with adaptability, facilitating the selection, and refinement of the motor solutions according to environmental demands (Bartlett et al., 2007). Specifically, this mechanism would promote the exploration of redundancy within the motor system and ease motor adjustments. Consequently, the examination of motor system variations has captivated the interest of several researchers, with investigations focusing on the role of variability according to stages of learning (Bernstein, 1967; Latash, 2010), as well as striving to identify the optimal level of variability for optimizing the adaptation process (Harbourne and Stergiou, 2009).

Variability plays a crucial role in the learning process, not only in motor skills but also in cognition, as highlighted in the manuscript by Zhang et al.. However, its effects are not uniform across all the studies. Instead, findings suggest that depending on which condition variability is measured and quantified, it can be related to different changes in long-term performance. A previous step to clarify the underlying rationale for the controversial results is to enhance the precision and accuracy of the terminology used in this field. The paper by Hossner and Zahno defines concepts such as variance, variability, exploration, exploitation, noise, error, and how they are linked to motor control and learning. Future research should homogenize those terms to ease the understanding of how motor variability serves a purposeful role in shaping motor control and facilitating learning.

Another aspect that should be standardized is the way motor variability is measured. There are numerous linear and non-linear methods to compute motor variability. Some works have already pointed out the limitations of traditional linear methods in capturing the intricate characteristics of human movement (Caballero et al., 2014). Non-linear tools seem to offer a more comprehensive approach by examining the non-linear relationships, temporal dynamics, and emergent properties of movement. Thus, implementing non-linear analysis techniques such as entropy measures (Anderson et al.; Aniszewska-Stȩpień et al.) or autocorrelation analyses (Kirchner et al., 2014; Barbado Murillo et al., 2017) reveal a valuable method for capturing the complexity and dynamic of human motor behavior.

Finally, current investigations have highlighted that the impact of motor variability on motor control and learning processes depends on two key factors: the nature of the task being performed and the capabilities of the individual involved.

Different tasks present distinct demands and constraints, which can either benefit from or be hindered by motor variability. For example, tasks that require precise and consistent movements, such as archery, may benefit from reduced motor variability to achieve high accuracy (Churchland et al., 2006; Shmuelof et al., 2012). On the other hand, tasks involving exploration or adaptation, such as learning a new movement pattern, may benefit from increased motor variability as it allows for greater exploration of movement possibilities (Riley and Turvey, 2002; Davids et al., 2003).

Individual capabilities, including motor skill level and adaptability, also influence the role of motor variability. Highly skilled individuals may exhibit lower motor variability due to their ability to produce more consistent and refined movements. In contrast, individuals with limited motor control or learning abilities may benefit from increased motor variability as it provides opportunities for exploration and learning (Remec et al.).

There is a need to understand how motor variability interacts with these factors and its implications for designing effective training programs and interventions tailored to individuals' needs. Works like the one proposed by Aniszewska-Stepień et al. are examples of how the level of learning varies according to the level of variability induced by the practice conditions. From the editors' point of view, these works also highlight the path that future investigations should follow: more research is needed to address which is the optimal variability load that should be promoted by the manipulation of practice conditions to foster motor learning according to each person's features.

## Author contributions

FM: Conceptualization, Supervision, Writing—review and editing. CC: Conceptualization, Resources, Writing—original draft, Writing—review and editing. DB: Conceptualization, Project administration, Resources, Writing—review and editing.
